# The accumulation of exosome-associated microRNA-1246 and microRNA-150-3p in human red blood cell suspensions

**DOI:** 10.1186/s12967-021-02887-2

**Published:** 2021-05-27

**Authors:** Yujie Kong, Xue Tian, Rui He, Chenyue Li, Haixia Xu, Li Tian, Zhong Liu

**Affiliations:** 1 Clinical Transfusion Research Center, Institute of Blood Transfusion, Chinese Academy of Medical Sciences and Peking Union Medical College, 26 Huacai Rd, Longtan Industry Zone, Chenghua District, Chengdu, 610052 Sichuan Province People’s Republic of China; 2 Key Laboratory of Transfusion Adverse Reactions, CAMS, 26 Huacai Rd, Longtan Industry Zone, Chenghua District, Chengdu, 610052 Sichuan Province People’s Republic of China

**Keywords:** Exosome, MicroRNA, Red blood cell (RBC) suspensions, Transfusion-related immunomodulation (TRIM)

## Abstract

**Background:**

Transfusion-related immunomodulation (TRIM) can be caused by exosomes, in which case, microRNAs (miRNAs) are one critical factor impacting exosome behavior. This study aims to investigate and analyze the expression profiles of exosomal miRNA in red blood cell (RBC) suspensions during storage and to identify potential TRIM-related miRNAs as well as their potential functions.

**Methods:**

A total of 25 packs of RBC suspensions were randomly collected. Exosome were extracted by ultracentrifugation and then identified and characterized by nanoparticle tracking analysis (NTA), transmission electron microscopy (TEM) and western blot (WB). Exosomal miRNA profiles were acquired using gene chips in five packs on week 1 and week 5. The expression data were compared from the two time points identifying accumulated miRNAs with statistical significance and their predicted targeting genes were analyzed. Based on the gene chip results, quantitative reverse transcription-polymerase chain reactions (qRT-PCR) were performed to verify miRNA accumulation in the rest 20 packs sampling on week 1, 3 and 5.

**Results:**

Gene chip analysis revealed that most exosomal miRNAs were enriched as the storage period progressed. Compared to samples from week 1, week 5 samples exhibited a total of 539 differential miRNA expressions, among which, 159 were statistically significant (*P* < 0.05) and 148 (93.08%) were accumulated. In the bioinformatics functional analysis, significant immunoregulatory annotations related to the thyroid hormone, mitogen-activated protein kinase (MAPK), focal adhesion and RAS signaling pathways were identified. The top 17 differential expression miRNAs were validated by qRT-PCR. The results confirmed that all the 17 miRNAs were accumulated with increasing storage time. In particular, miRNA-1246 and miRNA-150-3p were the most enriched strands by more than 150-folds in the 5-week storage period.

**Conclusions:**

As storage progressed, numerous exosomal miRNAs accumulated in the RBC suspensions, which are informatically connected to multiple immuno-signaling pathways. MiRNA-1246 and miRNA-150-3p may be essential mediators impacting the immunoregulation functions of exosomes in RBC suspensions, considering their significant accumulating scales. Further research should therefore focus on the relationship between these miRNAs and TRIM.

**Supplementary Information:**

The online version contains supplementary material available at 10.1186/s12967-021-02887-2.

## Background

Red blood cell (RBC) suspension is commonly transfused in clinic for patients suffering from anemia. It can be stored for 35 days at 4 ℃ in an additive solution with A-form of acid–citrate–dextrose (ACD-A). The storage time can be further extended to 42 days with saline adenine glucose mannitol (SAG-M) [1]. During storage, RBCs undergo a series of morphological and biochemical changes known as RBC storage lesions, which affect the quality and functions of RBCs [2]. The impact of the transfusion of RBCs with different storage period on individuals’ clinical outcomes has always been an active and controversial topic in the clinical community [3]. Studies have reported that prolonged RBC storage may increase the occurrence of complications and adverse clinical outcomes. Czubak-Prowizor et al. [4] reported that patients with cardiovascular disorders who received “older” RBC transfusions may suffer thrombotic complications. For trauma patients, prolonged RBC storage may increase the risk of deep vein thrombosis and bacterial infections [5, 6]. These complications are considered to be related to the changes in both RBCs and bioactive substances accumulated during storage [7].

Transfusion-related immunomodulation (TRIM) emerged as a potential explanation for these clinical complications. TRIM refers to certain immune-related responses caused by blood transfusion, some of which can affect the progression and prognosis of an existing disease. TRIM was first described as an immunosuppressive function in renal transplantation patients receiving perioperative blood transfusions [8]. TRIM was subsequently reported to be associated with increased tumor incidence, pathogen infection and mortality [9–11]. Adverse clinical outcomes caused by transfusing long-time stored RBCs have also been reported to be related to TRIM. The mechanisms of TRIM, however, remain largely unknown, and this is especially true of how stored RBC transfusions interact with the recipients’ immune system and what are the regulating cascades post transfusion [12].

Exosomes, a type of bioactivity mediator, are consistently produced throughout RBC suspension storage, which function as a tool of intercellular communication and molecule transport [13]. The generation of exosomes is thought to be related to TRIM in blood recipients [14]. Studies have reported that exosomes from RBC suspensions are capable of promoting inflammatory cytokine secretion and mediating T-cell proliferation responses [15]. Understanding the mechanism of RBC suspension-derived exosomes involved in TRIM, however, remains a challenge that requires further investigation. Previous research on exosomes mainly focused on their protein content. Recently, exosomal miRNAs have caught wide attention for their regulatory functions in gene expression, such as their ability to inhibit the translation of target genes [16]. MiRNA can be encapsulated in exosomes and stably exists in the recipient’s circulatory system. These exosomes can also fuse to recipient cells delivering exogenous contents, such as miRNAs [17]. MiRNAs act as crucial regulators in many biological processes, such as cell differentiation, signal transduction and immunomodulation [18].

This study focuses on the miRNA contained in exosomes and intends to analyze the dynamic alterations in the expression of exosomal miRNA in RBC suspensions during the storage period. A following bioinformatics analysis was conducted to predict the potential functions associated with immunoregulation in predominantly accumulated miRNAs. Further in vitro and in vivo studies will be performed to investigate the impact of the most accumulated miRNA on TRIM.

## Materials and methods

### Study samples

Twenty-five packs of randomly selected non-leukoreduced RBC suspensions with anticoagulants (ACD-A) were obtained at the Central Blood Bank of Deyang City, Sichuan province, China. Exosomes extracted from five packs underwent gene chip analysis while the rest exosome samples were collected for quantitative reverse transcription-polymerase chain reaction (qRT-PCR) validation based on gene chip analysis results. This research protocol was approved by the ethics committee of the Institute of Blood Transfusion of the Chinese Academy of Medical Sciences.

### RBC suspension storage, exosome isolation and purification

RBC suspensions were split into 100 mL aliquots in 100-mL transfer bags and stored at 4 °C. The aliquots were then subjected to exosome extraction at week 1, 3, and 5 during storage. The aliquots were first centrifuged at 3000*g* for 20 min to obtain supernatants. Exosomes were isolated from the supernatants by ultracentrifugation. The protocol includes a 10-min initial stage at 300*g*, a second 10-min stage at 2000*g*, a 30-min supernatant separation stage at 10,000*g* and a final 70-min exosome separation stage at 1,00,000*g* for twice. The exosome precipitate was resuspended in 200 µL of phosphate-buffered saline (PBS). Then, 0.22-μm centrifugal filters (Millipore, USA) were applied to remove large extracellular vesicles (EVs).

### Exosome identification

The extracted exosomes from RBC supernatants were verified by nanoparticle tracking analysis (NTA), transmission electron microscopy (TEM) and western blot analysis (WB).

The particle size distribution was measured by NTA using the NanoSight NS300 instrument (Malvern Instruments, UK). The sizes of the particles were analyzed using DTS v5.10 software (Malvern Panalytical, UK), and the results are presented in a particle size distribution graph.

For TEM analysis, copper grids were placed in exosome suspensions and fixed by 2% paraformaldehyde overnight. The morphology of the isolated exosomes was captured by TEM (FEI Tecnai™ G2 Spirit, Czech Republic) at 80 kV.

In WB analysis, exosomes were first treated with a RIPA lysis buffer, and the protein concentration was calculated using the bicinchoninic acid (BCA) method. Absorbance values were detected by Varioskan LUX (Thermo Fischer Scientific, USA). Exosome protein markers against TSG101 (Abcam, UK), CD9 (Abcam, UK) and CD63 (Santa Cruz, USA) were validated via WB analysis. Protein markers against Calnexin were also detected as a negative control. Lysates of Hela cells were tested as control samples. Signals of the membranes were captured and imaged by ChemiScope Mini 3000 (CLINX, Shanghai).

### RNA isolation and cDNA preparation

The Total RNA was extracted from exosomes using the QRIzol reagent and the miRNeasy Mini Kit (Qiagen, Germany). The quantity and quality of the total extracted RNA were measured by using the Agilent 4200 platform and the Qubit 2.0 Fluorometer (Thermo Fisher Scientific, USA). Only high-quality RNA samples were used for subsequent experiments (total RNA ≥ 0.5 μg, OD_260/280_ = 1.9–2.1 and RNA concentration ≥ 20 ng/μL). The miScript II RT Kit with a miScripthispec buffer (Qiagen, Germany) was used to prepare cDNA for subsequent gene chip and qRT-PCR validation.

### MiRNA profiling

Microarray analysis was performed by Gminix Biotechnology Company (Shanghai, China) using the Affymetrix Gene Chip miRNA 4.0 array (Affymetrix, USA). Gene chips were then scanned using the Gene Chip^®^ Scanner 3000 7G (Affymetrix). CEL-files of the raw data were exported and then uploaded to the Gminix-Cloud Biotechnology Information (GCBI) website of Genminix Informatics Co., Ltd. (Shanghai, China; http://www.gcbi.com.cn) for further analysis. The data were analyzed applying the robust multichip analysis algorithm (RMA) with Affymetrix default analysis settings and global scaling normalization. The successive approximation method (SAM) was then used for differentially expressed miRNA analysis. According to the filter condition |fold change| > 1.2 and *P* < 0.05, the final differential results were generated.

### Bioinformatics analysis of differentially accumulated exosomal miRNA

The target genes of various differentially increased exosomal miRNA were predicted by miRBase and TargetScan. A gene ontology (GO) analysis of target genes was performed using *P* < 0.05 to define statistically enriched GO categories. Pathway analysis was used to determine the significant pathways of the differential genes according to the Kyoto encyclopedia of genes and genomes database (http://www.genome.jp/kegg/). A miRNA–genes network was built to show the interactions of various strands of miRNA with their target genes.

### Identification of miRNA expression by qRT-PCR

The gene chip results suggested 17 top miRNAs that were accumulated more than ten-fold at week 5 compared to week 1 of storage time. Their expression changes in the rest 20 packs were validated by qRT-PCR. qRT-PCR was performed following the manufacturer’s miScript SYBR PCR kit (Qiagen, Germany) protocol with different miRNA primers for each cDNA sample of reverse transcription. The miRNA qPCR primers were synthesized by RIBOBIO Biotech (Guangzhou, China). And Cel-miRNA-39 (Qiagen, Germany) was used to normalize potential sample-to-sample variations and technical variations. Relative miRNA changes were calculated using the $2^{{ - \Delta \Delta {\text{CT}}}}$ method.

## Results

### Analysis and characterization of exosomes derived from RBC suspensions

**Fig. 1:**
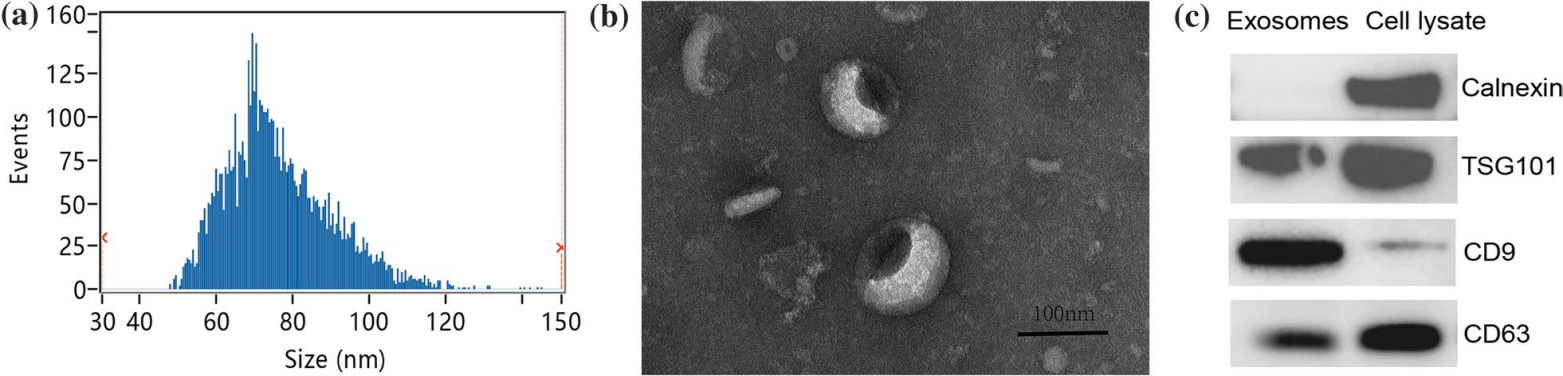
Characterization of exosomes isolated from RBC suspensions at week 5. **a** NTA for the particle size distribution of exosomes. **b** TEM for the morphology of exosomes. Scale bar, 100 nm. **c** WB identified the specific immunological markers TSG101, CD9 and CD63 of the exosomes

NTA, TEM and WB were used to identify and analyze the characterization of exosomes isolated from RBC suspensions. NTA particle size distribution analysis demonstrates that the isolated vesicles at week 5 presented the typical exosome size with particle diameters ranging from 30 to 150 nm (Fig. 1a). The average diameter of the articles was 76.07 ± 13.65 nm. More than 95% of the particles falls below the 120 nm diameter range, and 99.98% were below 150 nm. TEM confirmed the exosome morphology to be cup-shaped particles (Fig. 1b). Exosomal surface markers CD9, CD63 and TSG101 were tested positive in WB with negative Calnexin marker readings (Fig. 1c).

### Comparison of differentially accumulated exosome-associated microRNA during RBC suspension storage

**Fig. 2:**
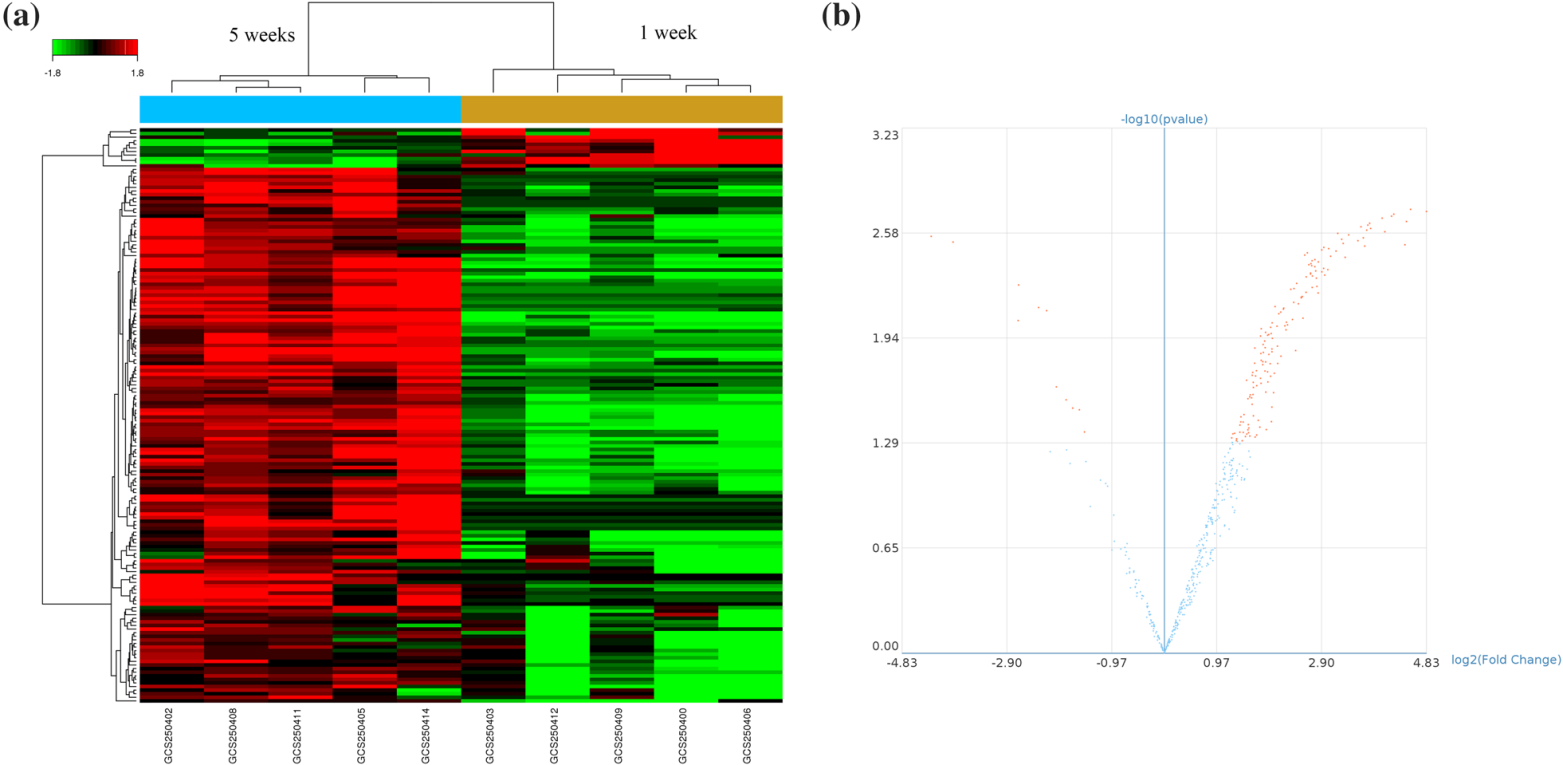
Microarray assay of differential miRNA in exosomes derived from RBC suspensions. **a** Heatmap of differential miRNA expressed in exosomes stored for week 5 vs. week 1. Each column represents an RBC suspension sample, and each row represents a kind of miRNA. The color describes the expression level of each miRNA in each sample, where red indicates a high level of expression and where green indicates a low level of expression. **b** Volcano plot shows the distribute expression of differential miRNAs. Different colors correspond to the levels of expression, where red represents significantly differential miRNA (*P* < 0.05)

The gene chip identified a total of 539 miRNAs during the 5-week storage. Figure 2a and b demonstrate information regarding group-specific signal intensities of the exosomal miRNA profile and the volcano plot between week 1 and week 5. Statistical analysis revealed 159 miRNAs changed differentially (148 increased and 11 decreased, |fold change| > 1.2 and *P* < 0.05), 59 of which were accumulated more than fivefold at week 5 compared to week 1. Table 1 and Additional file 1: Table S1 list the mean signal, fold changes and *P* values of the 59 miRNAs at week 1 and week 5.

**Table1:** List of differential exosomal miRNAs accumulated more than tenfold at week 5 vs. week 1 storage time of RBC suspensions

miRNA	week 5 log_2_ mean signal	week 1 log_2_ mean signal	week 5 vs. week 1 fold change	*P* value
hsa-miR-6824-5p	6.3266	1.7901	23.2080	0.0019
hsa-miR-6716-5p	7.7924	3.3319	22.0159	0.0022
hsa-miR-1246	8.4004	3.9677	21.5962	0.0031
hsa-miR-939-5p	6.9238	2.6953	18.7462	0.0020
hsa-miR-4433-3p	7.4835	3.2970	18.2076	0.0021
hsa-miR-4701-3p	6.6046	2.5412	16.7191	0.0026
hsa-miR-6849-5p	5.9334	1.9205	16.1438	0.0021
hsa-miR-595	5.7246	1.9235	13.9395	0.0024
hsa-miR-150-3p	6.0808	2.2974	13.7697	0.0023
hsa-miR-7844-5p	5.6850	1.9356	13.4488	0.0023
hsa-miR-6798-5p	8.3634	4.6340	13.2639	0.0025
hsa-miR-4689	7.9570	4.2640	12.9339	0.0031
hsa-miR-1180-3p	6.1168	2.4509	12.6924	0.0028
hsa-miR-3064-5p	5.4298	1.8060	12.3269	0.0024
hsa-miR-1225-5p	6.5773	3.0099	11.8550	0.0029
hsa-miR-6819-5p	5.6768	2.2801	10.5317	0.0027
hsa-miR-1268b	7.3709	4.0387	10.0714	0.0037

### Bioinformatic analysis: GO function enrichment and KEGG pathway of predicted target genes of significantly differentially accumulated miRNAs

To further determine the functions of the significantly accumulated miRNAs, the miRanda and Targetscan databases were used to predict targeting genes of these miRNAs, suggesting 5538 non-repeated genes.

**Fig. 3:**
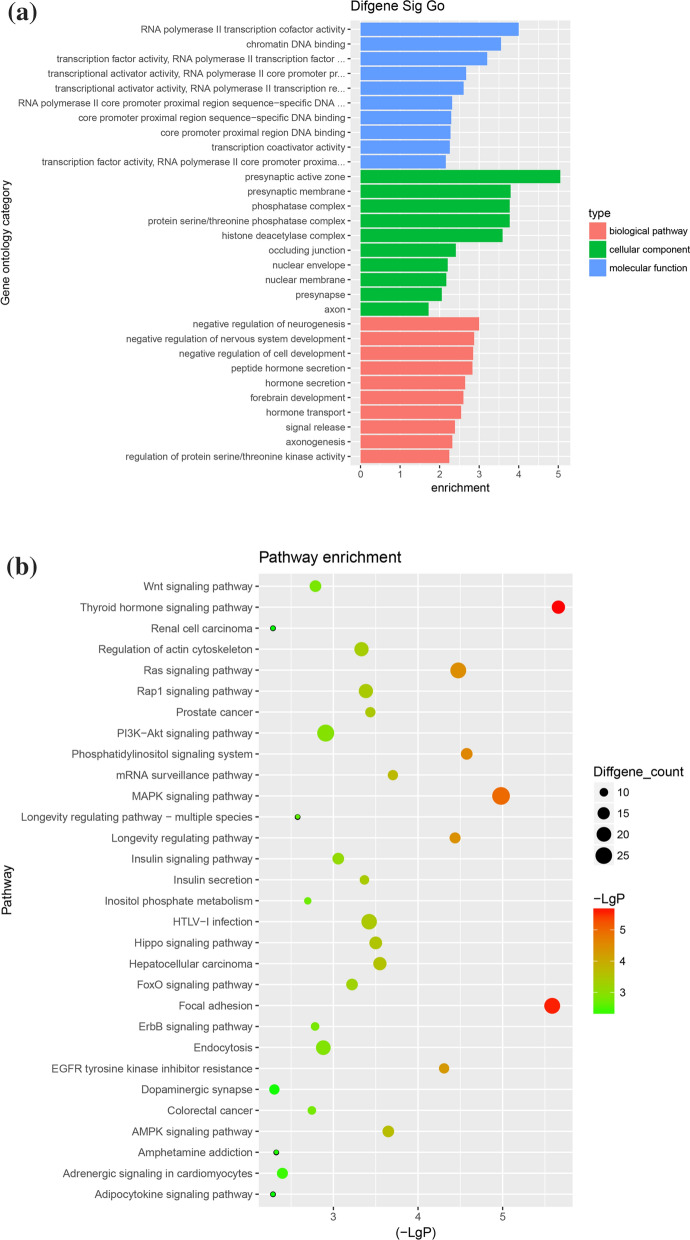
Prediction of the functions and signal pathways of target genes for significant accumulated exosomal miRNA via GO and KEGG pathway analysis. **a** The GO function enrichment terms of target genes for the significantly accumulated miRNA sat week 5 vs. week 1 of storage time; the function terms were ranked from high to low by enrichment score. **b** The KEGG pathway analysis of the target genes for the significantly accumulated miRNAs at week 5 vs. week 1 of storage time. The dot color represents −log (*P* value), and the dot size represents the number of target genes included in the pathway

**Fig. 4:**
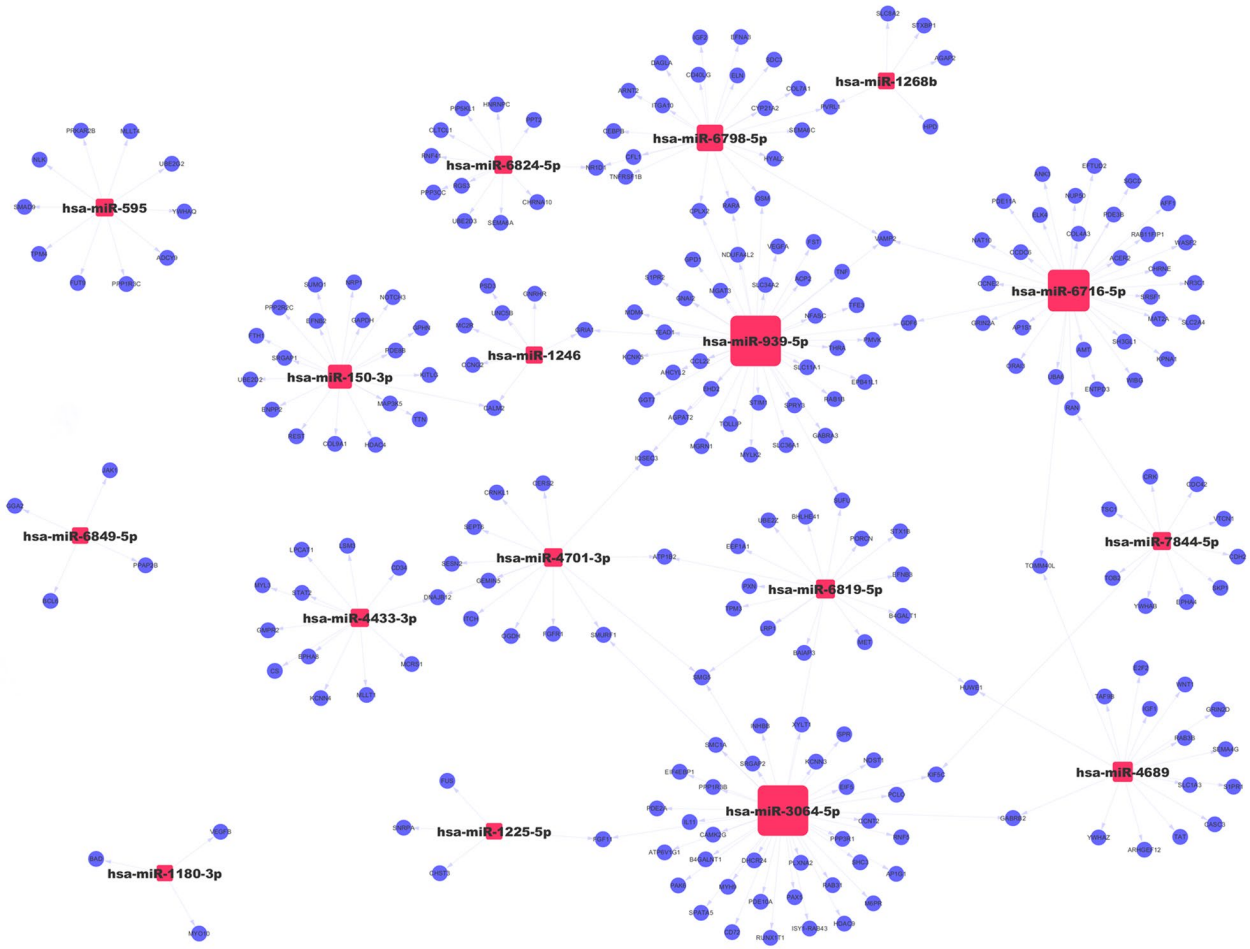
The networks of the top 17 exosomal miRNAs that accumulated more than tenfold in week 5 vs. week 1 of storage time, along with the target genes

GO analysis described the function of the predicted targeting genes, as the GO database mainly includes biological pathways, cellular components, and molecular functions. The results of GO overexpression analysis indicate that the genes were primarily involved in RNA polymerase II transcription cofactor activity, chromatin DNA binding, transcription factor activity and RNA polymerase II transcription factor binding (biological pathways). They were also primarily involved in the presynaptic active zone, the presynaptic membrane and the phosphatase complex (cellular components) as well as in the negative regulation of neurogenesis, the negative regulation of nervous system development and the negative regulation of cell development (molecular functions) (Fig. 3a). KEGG pathway analysis shows that the most significant pathway was the thyroid hormone signaling pathway (Channel ID: 04919), which was correlated with growth, development and metabolism. The target genes of the differential miRNA, however, were mainly enriched in the MAPK signaling pathway (Path ID: 04010), which played important roles in various cellular functions (Fig. 3b). Other pathways were significantly enriched, including focal adhesion (Path ID: 04510) and the Ras signaling pathway (Path ID: 04014). Figure 4 describes the network of the top 17 accumulated miRNAs, together with their targeting genes.

### Verification of miRNAs expression by qRT-PCR

The expression trend of the top 17 miRNAs were validated by qRT-PCR at week 1, 3 and 5, in 20 of the 25 packs of RBCs. The results are presented in relative expression changes, confirming all 17 miRNAs were increased across storage period. From week 1 to week 3, 10 miRNAs accumulated statistically significant, among which miRNA-1246, miRNA-150-3p and miRNA-3064-5p increased more than tenfold. The most abundant miRNA at week 3 was miRNA-150-3p with an average change of 73.67-fold against week 1 background.

A total of 11 miRNAs accumulated significantly from week 3 to week 5, among which miRNA-1246 exhibited the most change.

**Fig. 5:**
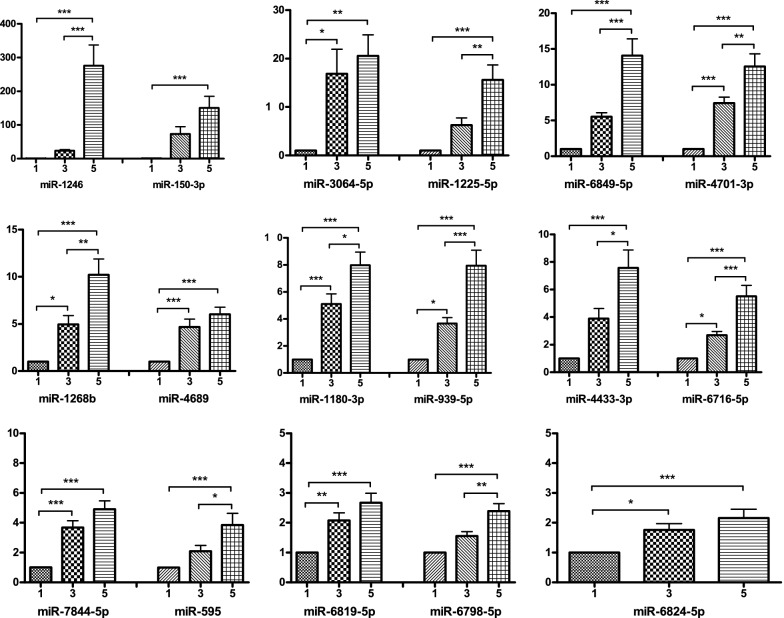
Validation of exosomal miRNA accumulated more than tenfold at week 5 vs. week 1 storage time (*P < 0.05, **P < 0.01 and ***P < 0.001). The horizontal axis represents storage time, with week 1 at 1, week 3 at 3 and week 5 at 5. The vertical axis represents relative expression changes

Comparing data at week 5 and week 1, all candidates experienced statistically significant changes (*P* < 0.01). The average changes in six miRNAs were more than tenfold: miRNA-1246, miRNA-150-3p, miRNA-3064-5p, miRNA-1225-5p, miRNA-6849-5p and miRNA-4701-3p (high to low in relative expression changes). MiRNA-1246, miRNA-1225-5p and miRNA-6849-5p were predominantly accumulated at week 5. Among these miRNAs, there were two that accumulated by more than 150-folds at week 5 compared to week 1. On average, miRNA-1246 increased 275.94-folds, and miRNA-150-3p increased 150.93-folds (Fig. 5).

## Discussion

Despite RBCs’ rapid application in clinical transfusion, they may raise adverse reactions in recipients, such as acute hemolytic transfusion reactions and delayed hemolytic (or serologic) transfusion reactions [19]. Sufficient evidence now supports the connection between RBC transfusion and immunomodulation via various mechanisms. The recent interest in the relationship between TRIM and extracellular vehicles from blood components has raised heated discussion in the academic community [20, 21]. Some studies suggest that exosomes in the RBC units may promote TRIM by pro-inflammatory cytokines and immune activation [12, 15]. However, such notion requires further illumination. Recent publications suggest a range of miRNAs in exosomes are involved in the regulation of immunity, including the innate immunity, the development of T and B cells, and proliferation of monocytes and neutrophils [22, 23]. In this study we sought to explore the exosomal miRNAs as potential functional molecules impacting TRIM. The miRNA profiles of supernatant exosomes from non-leukoreduced RBC suspensions were characterized and analyzed across a 5-week storage period and the significantly accumulated miRNAs were identified and annotated according to the function of their predicted targeting genes.

One similar study analyzed the miRNA profiles of exosomes collected from leukoredced RBC units in three separate samples obtained on day 14 of storage [24]. Compared to the in-data-base (miRDeep) 287 miRNAs previously reported in mature erythrocytes [25], the cross results revealed that 78 miRNAs were both expressed in all exosome samples and mature erythrocytes. The top three enriched miRNAs were miRNA-125b-5p, miRNA-4454 miRNA-451a, and these miRNAs were validated by qRT-PCR. The results show higher abundance of miRNA-451a than others and the expression increased with extending storage time, on average, 7.58-folds of increase across a 5-week storage period. This result is different from ours, which may be due to its comparison of miRNA expression between single miRNA profiling time point at day 14 and the previous identified miRNA in mature RBC. Our study, on the other hand, set different profiling points across the 5 weeks storage, ensuring the screening of miRNA with constant increase. Notably, we’ve identified several miRNAs with changing accumulation curves, further addressed the notion that single profiling time point may limit screening results.

In addition, our study identified miRNAs with a larger increase scale at more than 150-folds. This may be caused by the different selection of sampling source. We adopted non-leukoreduced RBC suspensions as the exosome source, considering its predominant occupation of the clinical transfusion market. In this sense, the exosome-associated miRNAs profiled in this study may not be limited to single RBC source but also other cells in the suspension. We predict the most enriched exosomal miRNA across the storage period might impact the quality of RBC suspensions before transfusion. This notion, however, requires further investigation.

GO and KEGG were used to predict the potential functions of the target genes of the statistically abundant types of miRNA within 5 weeks of storage time. The results indicate that the encoded proteins mainly came from synapse-related proteins and phosphatase complexes (presynaptic active zone, presynaptic membrane, phosphatase complex and protein serine/threonine phosphatase complex) that had transcription factor activities and DNA binding functions involved in the development of different systems, hormone secretion and transportation. The KEGG database was used to further annotate the target genes, revealing significant enrichment in the thyroid hormone signaling pathway. The thyroid hormone is closely correlated with immunoregulation and plays an essential role in several inflammatory-related processes, such as the production of cytokines and chemokines [26]. The MAPK signaling pathway experienced the greatest enrichment of its target genes. The MAPK signaling pathway contains three main kinases, namely the MAPK kinase kinase, the MAPK kinase and MAPK. They activate and phosphorylate downstream proteins to regulate and control various physiological and pathological functions, including innate immunity, inflammation, apoptosis, cell growth, cell differentiation, tumor invasion and metastasis, among others [27, 28]. In addition, focal adhesion and the Ras signaling pathway were also significantly enriched, both of which play important roles in immunomodulation. Focal adhesion participates in multiple biological processes including cell proliferation and the regulation of gene expression, and it serves as a scaffold for many signaling pathways [29]. The Ras protein with GTPase activity acts as a switch protein in the cell signaling pathway, regulating cell differentiation, proliferation, apoptosis and survival, among other activities [30]. Recent research has revealed that RAS plays a vital role in the formation of exosomes, and it may play a signaling role through exosome secretion [31]. The target genes of miRNAs accumulated during storage were significantly enriched in the above signaling pathways, and these signaling pathways played important roles in the immune system. Therefore, by regulating these signaling pathways, the exosomal miRNAs enriched in old RBC suspensions could be involved in immunoregulation.

RBC suspensions stored for 5 weeks had 17 significantly different miRNAs accumulated more than tenfold compared with those of RBC suspensions stored for 1 week. Afterward, qRT-PCR identification results show that there were six miRNAs accumulated more than tenfold at week 5 compared to week 1 storage. The six most accumulated miRNAs are partly reported to play potential roles in immuno-regulation. For instance, in dilated cardiomyopathy (DCM), miRNA-3064-5p, miRNA-6849-5p and miRNA-4701-3p are upregulated in abnormal activated CD4  + T cells, which may be associated with the proliferation of CD4  + T cells [32]. The Infusion of these exosomal miRNAs in long-time stored RBC transfusion may participate in the regulation of CD4  + T cell-mediated inflammatory and immune responses. Ranked by relative expression changes compared to week 1, miRNA-1246 and miRNA-150-3p were the two most abundant miRNAs both at week 3 and week 5. Exosome-derived miRNA-1246 in hypoxic glioma has been proven to be related to the induction of M2 macrophage polarization by targeting TERF2IP [33]. In addition, miRNA-1246 also mediates lipopolysaccharide (LPS)-induced lung injury and neutrophil activation by targeting angiotensin-converting enzyme 2 (ACE2) [34]. MiRNA-150-3p has been reported to play an important role in the anti-apoptosis process [35] and is also involved in inflammation pathways [34]. By inhibiting miRNA-150-3p expression, sacubitril/valsartan can effectively alleviate cyclophosphamide-induced lung inflammation [36]. Collectively, these two miRNAs (over 150-folds at week 5) are both associated with pro-inflammatory responses and might promote transfusion-related acute lung injury (TRALI). The transfusion of long-time stored RBC suspensions with these enriched exosome-associated miRNAs may have a potential role in individual immuno-regulatory responses. However, their associations with TRIM or transfusion-related adverse reactions still needs further investigation.

## Conclusions

This study revealed that many exosome-associated miRNAs existed in RBC suspensions and can accumulate during storage. Analysis of these exosome-associated miRNAs indicates that they may possess multiple functions during storage or post transfusion. The most enriched miRNA-1246 and miRNA-150-3p, in particular, might be essential mediators of immunoregulation. Following research on the mechanism of how these miRNAs impacting TRIM is to be conducted next.

## Supplementary Information

**Additional file 1: Table S1.** List of differential exosomal miRNAs accumulated more than five-fold at week 5 vs. week 1 storage time of RBC suspensions.

## Data Availability

The datasets used and/or analyzed during the current study are available from the corresponding author on reasonable request.

## Abbreviations

TRIM: Transfusion-related immunomodulation
RBC: Red blood cell
miRNA: MicroRNA
NTA: Nanoparticle tracking analysis
TEM: Transmission electron microscopy
WB: Western blot
qRT-PCR: Quantitative reverse transcription-polymerase chain reaction
MAPK: Mitogen-activated protein kinase
ACD-A: A-form of acid–citrate–dextrose
SAG-M: Saline adenine glucose mannitol
PBS: Phosphate-buffered saline
EV: Extracellular vesicle
BCA: Bicinchoninic acid
RMA: Robust multichip analysis
SAM: Successive approximation method
GO: Gene ontology
KEGG: Kyoto encyclopedia of genes and genomes
DCM: Dilated cardiomyopathy
LPS: Lipopolysaccharide
ACE2: Angiotensin-converting enzyme 2
