# Supplementary material for: The accumulation of exosome-associated microRNA-1246 and microRNA-150-3p in human red blood cell suspensions
Source: J Transl Med. 2021 May 27;19:225. doi: 10.1186/s12967-021-02887-2 (PMC8157439; doi:10.1186/s12967-021-02887-2)
Supplement: Supplementary file 1 — Additional file 1: Table S1. List of differential exosomal miRNAs accumulated more than five-fold at week 5 vs. week 1 storage time of RBC suspensions. [file 12967_2021_2887_MOESM1_ESM.docx]

**Table S1.** List of differential exosomal miRNAs accumulated more than fivefold at week 5 vs. week 1 storage time of RBC suspensions

| miRNA | week 5 log_2_ mean signal | week 1 log_2_ mean signal | week 5 vs. week 1  fold change | *P*-value |
| --- | --- | --- | --- | --- |
| hsa-miR-6824-5p | 6.3266 | 1.7901 | 23.2080 | 0.0019 |
| hsa-miR-6716-5p | 7.7924 | 3.3319 | 22.0159 | 0.0022 |
| hsa-miR-1246 | 8.4004 | 3.9677 | 21.5962 | 0.0031 |
| hsa-miR-939-5p | 6.9238 | 2.6953 | 18.7462 | 0.0020 |
| hsa-miR-4433-3p | 7.4835 | 3.2970 | 18.2076 | 0.0021 |
| hsa-miR-4701-3p | 6.6046  2.541192 | 2.5412 | 16.7191 | 0.0026 |
| hsa-miR-6849-5p | 5.9334 | 1.9205 | 16.1438 | 0.0021 |
| hsa-miR-595 | 5.7246 | 1.9235 | 13.9395 | 0.0024 |
| hsa-miR-150-3p | 6.0808 | 2.2974 | 13.7697 | 0.0023 |
| hsa-miR-7844-5p | 5.6850 | 1.9356 | 13.4488 | 0.0023 |
| hsa-miR-6798-5p | 8.3634 | 4.6340 | 13.2639 | 0.0025 |
| hsa-miR-4689 | 7.9570 | 4.2640 | 12.9339 | 0.0031 |
| hsa-miR-1180-3p | 6.1168 | 2.4509 | 12.6924 | 0.0028 |
| hsa-miR-3064-5p | 5.4298 | 1.8060 | 12.3269 | 0.0024 |
| hsa-miR-1225-5p | 6.5773 | 3.0099 | 11.8550 | 0.0029 |
| hsa-miR-6819-5p | 5.6768 | 2.2801 | 10.5317 | 0.0027 |
| hsa-miR-1268b | 7.3709 | 4.0387 | 10.0714 | 0.0037 |
| hsa-miR-3148 | 5.0453 | 1.7497 | 9.8194 | 0.0030 |
| hsa-miR-1224-5p | 4.9115 | 1.6434 | 9.6334 | 0.0028 |
| hsa-miR-4417 | 4.7814 | 1.5850 | 9.1671 | 0.0026 |
| hsa-miR-4433b-3p | 8.7068 | 5.5427 | 8.9642 | 0.0034 |
| hsa-miR-6782-5p | 6.3763 | 3.3114 | 8.3684 | 0.0036 |
| hsa-miR-6127 | 5.0514 | 2.0232 | 8.1578 | 0.0033 |
| hsa-miR-5195-3p | 4.7029 | 1.6900 | 8.0716 | 0.0044 |
| hsa-miR-1268a | 7.3690 | 4.4131 | 7.7597 | 0.0047 |
| hsa-miR-4322 | 4.5703 | 1.6310 | 7.6704 | 0.0032 |
| hsa-miR-1909-3p | 6.4341 | 3.4984 | 7.6510 | 0.0043 |
| hsa-miR-8089 | 5.7973 | 2.8943 | 7.4794 | 0.0045 |
| hsa-miR-3648 | 4.8948 | 1.9933 | 7.4719 | 0.0033 |
| hsa-miR-574-5p | 4.4224 | 1.5850 | 7.1476 | 0.0039 |
| hsa-miR-6802-5p | 6.7645 | 3.9302 | 7.1321 | 0.0061 |
| hsa-miR-3197 | 5.0004 | 2.1880 | 7.0244 | 0.0055 |
| hsa-miR-4440 | 4.8204 | 2.0191 | 6.9707 | 0.0049 |
| hsa-miR-3663-3p | 4.9899 | 2.2004 | 6.9141 | 0.0045 |
| hsa-miR-6808-5p | 4.7275 | 1.9522 | 6.8462 | 0.0038 |
| hsa-miR-4649-5p | 7.4336 | 4.6594 | 6.8411 | 0.0042 |
| hsa-miR-206 | 4.3141 | 1.5850 | 6.6306 | 0.0048 |
| hsa-miR-297 | 5.1536 | 2.4291 | 6.6094 | 0.0056 |
| hsa-miR-670-5p | 4.3078 | 1.5850 | 6.6015 | 0.0039 |
| hsa-miR-3149 | 4.3011 | 1.5850 | 6.5711 | 0.0041 |
| hsa-miR-6763-5p | 5.1502 | 2.4480 | 6.5080 | 0.0040 |
| hsa-miR-483-5p | 4.4704 | 1.7748 | 6.4784 | 0.0046 |
| hsa-miR-6752-5p | 9.9830 | 7.2890 | 6.4716 | 0.0047 |
| hsa-miR-5196-5p | 4.7928 | 2.1296 | 6.3346 | 0.0041 |
| hsa-miR-6871-5p | 4.3557 | 1.7197 | 6.2161 | 0.0034 |
| hsa-miR-7114-5p | 4.1999 | 1.5850 | 6.1260 | 0.0048 |
| hsa-miR-6856-5p | 4.3709 | 1.7587 | 6.1144 | 0.0060 |
| hsa-miR-6861-5p | 4.3169 | 1.7292 | 6.0116 | 0.0036 |
| hsa-miR-7107-5p | 6.6870 | 4.1313 | 5.8794 | 0.0087 |
| hsa-miR-6781-5p | 4.9498 | 2.4063 | 5.8303 | 0.0064 |
| hsa-miR-3679-5p | 4.9067 | 2.3821 | 5.7543 | 0.0065 |
| hsa-miR-3188 | 4.7797 | 2.2552 | 5.7534 | 0.0065 |
| hsa-miR-4793-3p | 4.7511 | 2.2808 | 5.5415 | 0.0071 |
| hsa-miR-1290 | 4.0241 | 1.5850 | 5.4232 | 0.0053 |
| hsa-miR-572 | 6.4109 | 3.9902 | 5.3546 | 0.0138 |
| hsa-miR-6870-5p | 4.2777 | 1.8887 | 5.2382 | 0.0057 |
| hsa-miR-3180 | 4.8989 | 2.5145 | 5.2213 | 0.0070 |
| hsa-miR-3180-3p | 5.6635 | 3.3025 | 5.1374 | 0.0089 |
| hsa-miR-7109-5p | 4.0591 | 1.7284 | 5.0304 | 0.0058 |
